# Patient experience of computerised therapy for depression in primary care

**DOI:** 10.1136/bmjopen-2015-008581

**Published:** 2015-11-30

**Authors:** Sarah E Knowles, Karina Lovell, Peter Bower, Simon Gilbody, Elizabeth Littlewood, Helen Lester

**Affiliations:** 1 Centre for Primary Care, NIHR School for Primary Care Research, University of Manchester, Manchester, UK; 2 School of Nursing, Midwifery & Social Work, University of Manchester, Manchester, UK; 3 Mental Health & Addiction Research Group, Department of Health Sciences, University of York, York, UK; 4 School of Health and Population Sciences, University of Birmingham, Birmingham, UK

**Keywords:** MENTAL HEALTH, PRIMARY CARE, depression

## Abstract

**Objective:**

To explore patient experience of computerised cognitive behaviour therapy (cCBT) for depression in a pragmatic randomised controlled trial (Randomised Evaluation of the Effectiveness and Acceptability of Computerised Therapy, REEACT).

**Design:**

Qualitative semistructured interviews with 36 participants.

**Participants:**

Depressed patients with a Patient Health Questionnaire 9 of 10 or above recruited into the REEACT randomised controlled trial.

**Setting:**

Primary care settings in England.

**Results:**

Participant experience was on a continuum, with some patients unable or unwilling to accept psychological therapy without interpersonal contact while others appreciated the enhanced anonymity and flexibility of cCBT. The majority of patients were ambivalent, recognising the potential benefits offered by cCBT but struggling with challenges posed by the severity of their illness, lack of support and limited personalisation of programme content. Low completion rates were commonly reported, although more positive patients reported greater engagement. Both positive and ambivalent patients perceived a need for monitoring or follow-up to support completion, while negative patients reported deliberate non-adherence due to dissatisfaction with the programme. Patients also reported that severity of depression impacted on engagement, and viewed cCBT as unsuitable for patients undergoing more severe depressive episodes.

**Conclusions:**

The study demonstrates both the unique demands and benefits of computerised therapy. cCBT was preferred by some patients and rejected by others, but the majority of patients were ambivalent about the therapy. cCBT could be offered within a menu of options in stepped care if matched appropriately to individual patients or could be offered with enhanced support to appeal to a greater number of patients.

**Trial registration number:**

ISRCTN91947481.

**Strengths and limitations of this study**

- The study was able to examine barriers and facilitators to engagement within the context of the REEACT trial, the largest independent study to date of computerised therapy.
- This is the first qualitative study of patient experience of computerised cognitive behaviour therapy (cCBT) for depression in the UK. The interview method enabled us to explore in detail both positive and negative factors impacting on patient experience of cCBT and how it was delivered in routine primary care.
- Patients received one of two specific programmes within the trial and results may not generalise to other computerised therapies. However, the trial examined specifically those programmes currently recommended for treating depression in the UK.
- Patients who would struggle most with cCBT may not have agreed to take part in the trial and so their views may be neglected. Furthermore, although the interview sample was representative of those involved in the trial as a whole, participants were predominantly White British and research is needed to explore the perspectives of other populations.

## Introduction

Depression is the leading cause of disability adjusted life years globally.^1^ The WHO recommends integrating mental health treatments into primary care in order to increase access to effective treatments.^2^ Alongside increasing prevalence, many economies are facing fiscal pressure that affect their health services. The struggle to meet the increasing demand for psychological therapies, particularly during financial downturns, has driven interest in how therapies can be delivered more efficiently and effectively.^3^ Computerised cognitive behaviour therapy (cCBT) is a rapidly advancing field that has been recommended within National Institute for Health and Care Excellence (NICE) clinical guidelines for depression.^4^ Proponents of cCBT have highlighted increased access for patients and the potential of these treatments to empower patients,^5^ ^6^ while others have been sceptical that therapy can work in the absence of a therapeutic relationship with a professional.^7^

Reviews of effectiveness have demonstrated that computerised therapy can improve depression outcomes, but there are also recognised problems with engagement and adherence,^8^ ^9^ with the significant drop-out rates leading to questions about whether cCBT can be considered an effective intervention in practice.^10^ Attention has therefore turned to examining the experience of patients using such programmes in order to better understand challenges to engagement and retention and also to understand the implementation of computerised therapies into practice. A growing number of studies have explored experience of using computerised therapy^11–14^ and have found evidence of both positive and negative attitudes. However, these studies have predominantly explored experience of cCBT provided with therapist support, rather than examining the experience of cCBT provided as a complete treatment without additional therapist input. Additionally, there have not been any studies exploring the experience of depressed patients recruited through health services in the UK. One study explored the experience of 18 Dutch patients with depression receiving cCBT^15^ and reported generally negative experiences, related to lack of support and lack of identification with content. However, some patients also highlighted the benefits of increased anonymity of cCBT. The present study is the first in the UK to recruit patients with depression from primary care and to explore patient experience of computerised therapy as provided within the context of a pragmatic trial.

Acceptability of an intervention to patients can have an impact on the clinical effectiveness of a treatment, affecting adherence and the ‘dose’ of therapy received.^16^ Studies of acceptability are critical to inform service delivery, consistent with policy recommendations to make patient experience central to the National Health Service (NHS),^17^ and to determine the place of cCBT in depression care in the UK. Qualitative methods have been recommended to explore acceptability in depth.^8^ The aim of the study was therefore to explore patient experience of cCBT in a sample of patients with depression treated in primary care, particularly focusing on engagement with the programme and acceptability of therapy delivered by computer without therapist support.

## Methods

### Sample

Participants in the REEACT trial (ISRCTN 91947481)^18^ were recruited through general practices (either through response to a postal invitation or directly referred during a GP consultation.) Eligibility criteria were a Patient Health Questionnaire (PHQ) 9 score of 10 or above, indicating a clinical level of depression and an ability to understand written English (as the cCBT programmes were both presented in English). All participants recruited had to be willing to try cCBT but it was not necessarily their preferred treatment—preference for cCBT or not was assessed at baseline with a single item question. For the present study, we aimed to interview a minimum of 30 participants and sought to sample participants based on expressed preference at baseline and engagement (self-reported number of modules of the programmes completed). In practice we adopted a convenience sampling method to ensure adequate recruitment rather than using an a priori sampling theme, and instead attended to preference and engagement in the topic guides and in analysis. The first 80 participants to complete their 4-month trial follow-up at each of the four research sites (Bristol, Manchester, Sheffield and York) were invited by letter to participate. Participants were then free to consider whether to take part and to contact the study team at their convenience. On contacting the study team, participants were given a full explanation of the study via telephone and the opportunity to ask questions or take further time to consider participation if desired. Once participants confirmed their wish to take part, interviews were then arranged at a time and place acceptable to the participant. Participants gave written consent to the researcher prior to start of the interview and were reminded of their right to withdraw at any time. Transcripts were anonymised prior to analysis by the study team to maintain participant confidentiality.

Full details of the main trial are reported in Gilbody *et al*.^18^ Ethical approval for the qualitative study was granted by Leeds East REC (08/H1306/77).

### Design

Semistructured interviews were conducted by three research associates at the participants’ homes. All had backgrounds in Psychology and had prior qualitative interview experience. Interviews lasted between 45 min and 1 h 30 min. Within the interviews, topic guides were used to suggest areas of discussion, informed by previous literature and the study aims, but we avoided using them as definite frameworks to limit or explicitly focus conversations. Topic guides included questions to explore both expressed preference (“Did you have a preference for cCBT before the trial, or not? Can you tell me more about that?”) and engagement (“How long did you use the programme for? What helped you to use it/why did you stop using it?”). Topic guides developed iteratively based on emerging themes, with later interviewees explicitly asked about perceptions of monitoring and support provided either by the trial team or by general practitioners (GPs), and explored contrasting perceptions of flexibility and autonomy. The study therefore involved elements of both inductive and deductive approaches, as we aimed to explore specific issues around delivery and acceptability, informed by existing research and by the aims of the study, but wished to remain open to novel or unexpected findings. This approach is consistent with other qualitative work exploring the acceptability of health technologies to patients.^19^ Both interviews and analysis were undertaken prior to the results of the trial being known. Interviews were conducted separately from any trial outcome assessments to avoid bias and to ensure that the interaction felt open and exploratory (in contrast to the predefined nature of the trial outcome assessments.) We assessed completed analysis of the interviews for data saturation (the point at which new categories or themes no longer emerged from further data^20^) in order to decide whether to initiate a second round of recruitment, which was agreed to be unnecessary.

### Analysis

Data were analysed using the constant comparative method as described by Boeije.^21^ All transcripts were analysed by SK, with a subset of 16 transcripts independently analysed by 3 of the other authors before consensus meetings to discuss all the transcripts collectively. Analysis consisted of three phases—data reduction through open coding of the transcripts, enabling immersion with the data and generation of initial codes based on significant or frequently occurring phrases, then data display through axial coding which developed the initial descriptive codes into key themes which reflected the overall constructs observed in the data. The final phase focused on conclusion-drawing, synthesising the findings into the core overarching themes which are presented here. Again, this analytical approach is consistent with prior research exploring patient experience of new health technologies.^19^ Overall analysis consisted of two key processes, fragmenting and connecting.^21^ Fragmenting refers to exploring the separate themes that emerge within each interview, to code items relevant to the individual research questions. Connecting emphasises the interview as a whole, to understand the themes together in context. These processes were conducted first within single interviews, then between interviews in the same group and finally across the sample as a whole. Disagreements on themes were discussed until agreement was reached.

## Results

Thirty-six patients were interviewed between January and October 2011, the point at which data saturation^20^ was judged complete. Thirty-four (94%) of the patients were White British, with 2 reporting Other White background. The mean age of patients was 51 with a range of 29–69, and 10 (28%) of the participants were male. The mean PHQ 9 at baseline was 19, with a range of 12–27. The sample was therefore representative of the wider trial population regarding gender and ethnicity, but the interviewed participants were on average older (51 years compared to 39 years in the trial overall).

Two thirds of the sample used the MoodGYM programme. Specific differences in themes between MoodGYM (MG) and Beating the Blues (BtB) programmes were not evident in the data and differences between the programmes did not appear to be associated with any differences in patient experience, and consequently we did not perceive it to be necessary to recruit further participants who had used BtB. Both programmes consist of modular ‘sessions’ lasting approximately 45 min, recommended to be completed at a rate of one per week (6 sessions for MG and 8 for BtB), which guide the user through cognitive behaviour therapy principles including interactive exercises and weekly ‘homework’ assignments to be completed between sessions.

Three key themes emerged from the data: acceptability; engagement and adherence; perceived absence of support. While patients varied in their reported acceptability, common issues relating to adherence and support were reported across the whole sample.

### Patient acceptability: perceived benefits and barriers

We observed in the data that participants could be broadly classified as ‘positive’, ‘negative’ or ‘ambivalent’ based on their perceptions of cCBT. Although it is not conventional to formally group qualitative respondents in this way, the simple categorisation adopted here may have utility in the context of this pragmatic assessment of health technology. Seventeen participants were categorised as ‘ambivalent’ (7 Beating the Blues, 10 MoodGYM), 10 were categorised as ‘negative’ (2 Beating the Blues, 8 MoodGYM) and nine as ‘positive’ (4 Beating the Blues, 5 MoodGYM). Patient characteristics and classifications are presented in table 1. Classifications were reached from consensus between the qualitative team based on rereading of the transcripts and identifying consistent differences in patient experience relating to their overall perception being positive, negative or ambivalent.

**Table 1:** Participant characteristics

Transcript code	Experience	Programme used	Site	PHQ 9 BL	Gender	Age	Ethnicity	Preference	Number of times used
DS0026	Ambivalent	Beating the Blues	York	26	M	57	1	No	4
DS0018	Ambivalent	Beating the Blues	York	17	F	53	1	No	7
DS0023	Ambivalent	Beating the Blues	York	19	F	46	1	Yes	6
DS0014	Ambivalent	Beating the Blues	York	13	M	54	1	Yes	8
DSB16.5	Ambivalent	Beating the Blues	Bristol	15	F	50	1	n/r	2
DSB0906	Ambivalent	Beating the Blues	Bristol	22	F	50	1	Yes	4
DS0019	Ambivalent	Beating the Blues	York	16	M	55	1	Yes	8
DS0024	Ambivalent	MoodGYM	York	26	F	43	3	Yes	6
DS0043	Ambivalent	MoodGYM	Sheffield	17	M	66	1	Yes	6
DS0015	Ambivalent	MoodGYM	York	19	M	48	1	Yes	18
DS007	Ambivalent	MoodGYM	Manchester	17	F	43	1	Yes	8
DS0013	Ambivalent	MoodGYM	York	21	F	48	1	Yes	1
DS0020	Ambivalent	MoodGYM	York	21	F	56	1	Yes	6
DSB2505	Ambivalent	MoodGYM	Bristol	23	F	32	1	Yes	6
DSB200511	Ambivalent	MoodGYM	Bristol	25	F	52	1	Yes	10
DS0012	Ambivalent	MoodGYM	Manchester	16	M	58	1	Yes	5
DS0025	Ambivalent	MoodGYM	Manchester	22	M	59	1	Yes	10
DS0029	Negative	Beating the Blues	York	13	F	54	1	Yes	2
DS003	Negative	Beating the Blues	Manchester	26	M	45	1	Yes	0
DS25511	Negative	MoodGYM	Bristol	12	F	65	1	No	8
DS004	Negative	MoodGYM	Manchester	25	F	29	1	No	5
DS008	Negative	MoodGYM	Manchester	14	M	32	3	Yes	1
DS0021	Negative	MoodGYM	York	15	F	56	1	No	4
DS006	Negative	MoodGYM	Manchester	24	M	59	1	Yes	2
DSB23.05	Negative	MoodGYM	Bristol	16	F	69	1	No	2
DSB0706	Negative	MoodGYM	Bristol	16	F	58	1	No	8
DS0011	Negative	MoodGYM	Manchester	21	F	59	1	Yes	0
DS0017	Positive	Beating the Blues	York	17	F	60	1	Yes	12
DS044	Positive	Beating the Blues	Sheffield	17	F	44	1	Yes	8
DS0016	Positive	Beating the Blues	York	24	F	48	1	No	5
DS0031	Positive	Beating the Blues	Manchester	15	F	40	1	No	8
DSB24.5	Positive	MoodGYM	Bristol	16	F	49	1	No	10
DS038	Positive	MoodGYM	Bristol	25	F	53	1	Yes	15
DSB240511	Positive	MoodGYM	Bristol	27	F	54	1	n/r	10
DSB0906	Positive	MoodGYM	Bristol	20	F	52	1	Yes	8
DS0045	Positive	MoodGYM	Sheffield	12	F	37	1	Yes	5

It is notable that these categorisations did not map clearly onto expressed prior preference for cCBT (this was discerned from a single item question at baseline which asked “Do you have a preference for receiving computerised therapy?”), with no consistent relationship between expressing a preference for cCBT or not at baseline and whether the participant consequently reported being positive or negative after experiencing the intervention (although numbers are too small to draw any definitive conclusions). This suggests participants may not be able to accurately predict whether the computerised therapy is appropriate for them based on initial preferences alone.

Extracts illustrating the perceived barriers and benefits are shown in table 2. Specifically, the four subthemes of Acceptability presented (‘Flexibility, ‘Autonomy’, ‘Relational’, ‘Connectedness’) illustrate how the same aspects of cCBT could be perceived both positively and negatively, depending on individual participant experience and preference.

**Table 2:** Extracts demonstrating how acceptability varied across a continuum

Continuum of experience	Flexibility	Autonomy	Relational	Connectedness
Negative	‘Too’ flexible—easy to avoid, difficult to sustain	Enforced autonomy—too demanding, felt like ‘work’	Lacks empathic response	Isolating, enhances feeling of loneliness
	“*When you've got your off days it's easier to not bother with the computer whereas, you know, if you've got a face to face it's not, to me, I think it's not polite to not turn up. So I think yeah it's definitely going to work, you know, against it being so flexible.”* (DS03, M, BtB)	“*To come and have to do something on a computer at night which I deemed as work, my mind automatically saw as work and effort…and the amount of motivation that it takes when you're depressed to go and do work it just doesn't seem to add up at all.” (*DS08, M, MG)	“*If you're feeling like that, then a computer telling you something isn't going to make any difference. Whereas somebody seeing you and seeing the state that you're in can make a big difference…, the verbal cuddle, which is what you need”* (DS11, F, MG)	“*You do feel very alone…[working on the computer] sort of highlights it”* (DS04, F, MG) “*I think maybe loneliness plays a part in it you know. So talking to the computer isn't going to be the…doesn't appear to be the answer”* (DS06, M, MG)
Ambivalent	Appreciated flexibility but greater monitoring/follow-up needed to support use	Interrupted autonomy—didactic, did not feel like it was user led	Lacks personalisation—too generic	Disconnection from characters
	“*I felt because the computer is here I thought it would be easier than going somewhere and seeing someone; but it didn't work out that way… I just sort of lost the oomph for it and then I kept forgetting to go on… I would have felt more obliged to do it if there was somebody keeping an eye on me” (*DS023, F, BtB)	“*I don't respond well to a computer saying go to the next page, go back to this page.”* (DS021, F, MG) “*There's certain parts of MoodGYM where the computer says, no, you know, and it was just so typical, I was just thinking, oh for goodness sake!…if you're wanting to get something done, don't put fifty obstacles in front of them getting the right answer, it's just daft.”(*DS025, M, MG)	“*I mean the CBT isn't really dealing with an individual. It's sort of very, very generic.”* (DS020, F, MG) “*Some of it didn't feel like it applied to me. I found it was most effective and most challenging when I read something and thought oh yes, that's me… just some way of putting in some basics about yourself at the beginning, which would either maybe shape or filter the content.”* (DS015, M, MG)	“*When they were relevant to me it was fine, you know, but when they weren't it was so frustrating”* (DSB0706, F, MG) “*That almost burst the bubble and broke the spell. I would step away from it and think no, that's not me,”* (DS015, M, MG) “*I think if you could have chosen your character was the most like you… because I had all young people who were just having boyfriend or shopping problems.”* (DS020, F, MG)
Positive	Patient controls when to use	Supported autonomy—empowering, encourages self determination	Appreciate anonymity or reduced pressure of not being face to face	Comforting—‘always there’
	“*When counsellors come they have to look at the clock when you're in the middle of bearing your soul the last thing you want is for someone to look at their watch. … with the computer you haven't got a time restriction have you?” (*DS038, F, MG) “*If I didn't feel like doing it today, I can leave until tomorrow, and nobody's going to say, ‘Oh you've cancelled your appointment, I can't see you tomorrow.’ It was there when I needed it”* (DS017, F, BtB) “*You could go back on yourself, you could go back and forward as much as you want, you could see what you'd put and what you were working towards, and that you could stop at any time if you wanted to and come back at a later time.”* (DS045, F, MG)	“*The way the computer was asking questions…it made you find in yourself how to reply to the questions.”*(DS016, F, BtB) “*Rather than just saying well here's your pills or sit there and talk to somebody for 35 minutes…actually felt like I was doing something to help myself.” (DSB24.5, F, MG)* “*I think if somebody said to me we could offer you some counselling, do you want a counsellor or a computer, then I think I would say computer …I mean I can talk for England, but actually doing it helps you think you're doing it for yourself as well, if you know what I mean. You're actually working it aren't you?”* (DS038, F,MG)	“*“[I felt] that the computer cared, and I know that sounds absolutely ridiculous, but it was like speaking to somebody but different. Maybe because I wasn't speaking to somebody, it didn't hurt me to write down my feelings” (*DS016, F, BtB) “*That quite appealed because I wanted to be private, I wanted to be in control”* (DS017, F, BtB) “*I think sometimes when you're sat in front of someone the words just dry up really …when you're sitting in front of someone. You feel like you're on the spot, you've got to make an answer straightaway.”* (DS045, F, MG)	“*I think that's really important with people when they're on the floor and you're there and you think you're the only one in the world and nobody's around, and you can get on there and, well, it's instant isn't it …if it's three o'clock in the morning, you just go straight to it and it's there”* (DS038, F, MG) “*If you were having a bit of a bad time and you couldn't sleep or something, and you felt that's what you needed to do, you could go on it at two o'clock in the morning if you wanted to. But I don't think a therapist is going to want you ringing up then! “* (DS045, F, MG)

Some patients found the absence of interpersonal contact with a therapist to be a fundamental problem and viewed therapy without face to face contact as ineffective. Others, by contrast, appreciated the enhanced privacy, flexibility and autonomy of working with the computerised programme. The majority of patients fell between these extremes, recognising the potential benefits but struggling with lack of adaptive content and also with the didactic presentation style within the programme. For those patients who reported ambivalent experiences, cCBT was not rejected outright but was considered ineffective without greater external support and frustrating due to the lack of personalisation of material within the programme. Technological difficulties in accessing the programme or dislike of computers, however, were not reported as barriers, which may reflect increasing familiarity with technology.

### Patient engagement and adherence

The acceptability continuum identified above did appear to impact engagement to some extent. Patients categorised as ‘positive’ showed a trend for greater adherence (as measured by self-reported number of times using the programme) and more often reported completing the programmes. On self-reported engagement, patients categorised as ‘positive’ reported using the programme an average of 9.1 times, while ‘ambivalent’ patients reported using the programme 6.7 times and ‘negative’ patients reported an average of 3.2 uses.

However, all patients reported some challenges to engagement and even ‘ambivalent’ and ‘positive’ patients reported some struggles to engage and not all the most positive patients reported completing the programme. These findings are consistent with the findings from the main trial, which demonstrate low levels of engagement and completion. While those patients who reported negative views of cCBT said they deliberately did not use the programme after the initial attempts, indicating poor engagement due to deliberate non-adherence, both ‘positive’ and ‘ambivalent’ patients reported intentions to adhere were disrupted by severity of depression and lack of follow-up. Some participants went further to suggest an iatrogenic risk of using the programme during severe episodes. Even when participants viewed the programme positively as a whole, they cautioned that the programme could be experienced as reflecting back their illness at them and that failure to cope with the demands of the programme could worsen the condition of already vulnerable patients:

The whole business about CBT is helping you to address the things but, you've got to be strong enough to address them because … you're opening up a Pandora's box. I was feeling so miserable that all those questions did was actually just hold the mirror and show me how miserable I was and, so, I just turned the programme off…And that was, for me, the first, kind of, two months really, three months, because I was just feeling so down (DS025, M, 59, MG)

If you're mildly depressed, or if you've turned the corner, then I think that's when it's appropriate. But I think if you were deeply depressed, and still struggling, then it would be much harder …I think you probably would fail and that would make you feel worse. Because the last thing you need is another failure when you're feeling really down. (DS017, F, 60, BtB)

This theme highlights how difficult therapy can be, with these demands exacerbated in self-help therapies where they must be managed solely by the patient.

The treatment is a test of strength…as in the commitment and the emotional strength, you know. Because thinking about your feelings and dissecting them, it is emotionally draining and it takes a lot out of you and while you're doing it can make you more upset. (DS04, F, 29, MG)

Patients indicated that the flexibility of cCBT could be a barrier to using it given the low motivation experienced by many people with depression and suggested that follow-up or monitoring may have improved adherence.

### Perceived absence of support

The REEACT trial provided supported cCBT, with technical support and general encouragement offered to trial participants on a weekly basis, for up to 8 weeks while they used the programme. An average of 3.1–3.3 calls were provided to patients as reported in the main trial paper, but structured psychological support was not provided. Participants perceived this to be inadequate to support engagement and considered their use of the programme to be unsupported, reporting a need for more motivational or emotional support to use the programme. Consistent with our focus on delivery of the intervention in routine primary care, we identified a consistent theme regarding perceptions of the role of GPs in providing such support. Participants did not consider that support for engaging with the package could be provided in routine primary care consultations, with monitoring provided by GPs considered important but either inappropriate or inadequate for meeting the perceived demands of the cCBT.

I think if they’d got the time you maybe would, but I think it’s indicative of the world we live in and the system that we live with. The good doctors are always in demand and you go back for your repeat prescription and you see who you see and there are maybe three of them that I would see, but you don’t see the one that suggested this therapy in the first place. So no, I’ve never talked about it to anybody. (DS029, F, 54, BtB)

Furthermore, a lack of time in the consultations and difficulties in providing continuity of care were frequently cited reasons for the lack of GP initiated follow-up from the patients’ perspectives:

I wouldn't have expected a GP to call me. It would have been nice, the family GP practitioner in the countryside but we live in a city and they've got what 10 000 patients on their book. So yeah I understand. (DS08, M, 32, MG)

What I'm talking about, he can't fit that in a ten minute GP slot at all, no, that's unfair on him, and it's also unfair on me (DS025, M, 59, MG)

All participants were therefore consistent in expressing a desire for greater support or monitoring but did not see their GP as being well placed to fulfil this role.

## Discussion

### Statement of principal findings

This study found that a subset of patients with depression treated in primary care experienced cCBT as fundamentally unacceptable. The benefits of therapy were considered inextricably linked with the experience of interpersonal support. For another group, cCBT provided an enhanced therapeutic experience precisely because it was not delivered through a traditional face to face encounter, but instead allowed enhanced accessibility, flexibility and anonymity. However, the majority of participants had a more mixed reaction to the technology, appreciating the potential benefits of cCBT but struggling with the content and delivery of the intervention. Critically, even when attitudes were ambivalent, poor adherence and engagement were typical and were attributed to severity of depression and absence of follow-up support to encourage adherence. There were no obvious differences in attitudes by gender or age, or according to the specific programme used.

Patients reporting positive experiences tended to report higher adherence and completion, but nevertheless expressed a desire for more substantial monitoring and follow-up to support engagement. Patients with a more negative experience reported deliberate non-adherence due to low satisfaction with the programme. Patients with ambivalent attitudes also reported that greater monitoring would have encouraged greater adherence. This suggests that more substantial support during provision could enhance the experience for those whose reaction was positive, through supporting completion, and would potentially promote the experience for those with more ambivalent attitudes to increase their engagement and satisfaction with the programme.

Our findings are consistent with other studies both in the UK and beyond. In Norway, Wilhelmsen *et al*^13^ found that persistence with computerised therapies was found to be higher when a feeling of ‘relatedness’ was met, which could be met through identification with the modules or through relating to a therapist who supported use of the programme. The present study similarly demonstrates that a sense of connection to the programme itself is possible for some patients but insufficient for others, who may instead need human support to achieve this sense of relatedness. In in an occupational sample in the UK, Schneider *et al*^22^ also observed that cCBT is actually preferred to face-to-face therapy for some people whereas others strongly prefer contact with a human therapist, and similarly recommended that attention be paid to both identifying which patients are most suited to computerised therapies (particularly considering which patients may require additional motivational or emotional support) and how such programmes can be improved to enhance patient experience or compensate for perceived limitations. The finding that GPs may not be best placed to provide follow-up support has also been observed outside the UK.^23^

### Strengths and weaknesses of the study

REEACT is the largest independent trial of cCBT yet to be conducted and enabled a detailed qualitative investigation of acceptability of cCBT in routine primary care, alongside a formal quantitative evaluation. It is also the first study of the acceptability of supported cCBT for patients being treated for depression in primary care in the UK. The data are limited to experience of two available programmes of cCBT, although lack of significant differences between the programmes suggests the themes may be generalisable across computerised therapy formats. The study found psychosocial barriers were more important to patients than structural barriers such as limited computer access or technological aptitude but this may be because patients experiencing these barriers self-select out of trials such as REEACT and their views may be under-represented. Participant response rate to the qualitative study was 10% across all sites except Sheffield where fewer patients were recruited. However, no obvious differences in views were observed across sites. The sampling strategy employed was opportunistic, and a more theoretical sampling strategy may have led to different observations. The sample was predominantly female and all participants were from a White background. This is consistent with the trial population as a whole, suggesting the sample is representative of the wider trial, although participants in the present study tended to be older. Further research is necessary to determine whether the results generalise to non-White backgrounds and to consider potential age differences in more depth.

### Implications of the study for clinicians and policymakers

The study examined the acceptability of cCBT as an intervention as delivered in routine care with technical/motivational support offered to participants. In a previous trial of cCBT using Beating the Blues,^24^ a nurse supported use by checking patients had logged on, had completed the session and scheduled the next session (no more than 5 min support per session). In the REEACT trial, support via telephone was offered after randomisation with no system of structured monitoring linked directly to the CBT content of the programmes. Our findings demonstrate that under routine conditions, adherence is low in the absence of structured monitoring and motivational support and follow-up monitoring are not routinely provided by primary care staff. A proportion of non-adherence may therefore be reduced by including more substantial monitoring protocols.^25^

The study highlights the demands made on patients who engage with psychological therapies, which may be exaggerated in cCBT where clinician input is minimised and the system places greater responsibility on individual patients to initiate, understand and continue with treatment. Some patients, however, reported preferring cCBT to traditional face to face therapies. It has been suggested that the key question is not whether cCBT works, but for whom. Our data suggest that depression care pathways need to be designed to be responsive to different patients’ experiences to ensure that patients best suited to cCBT are informed of this option. Such pathways should also adopt the principles of ‘stepped care’, with assessment of patient progress to rapidly identify those for whom the treatment is unacceptable after initial experience and identify alternatives, such as guided self-help or group therapies. This may be especially important given that preferences prior to treatment were not consistent with experience in practice in the present study. This would enable protocols to more effectively deliver cCBT and support use for patients who are ambivalent and at risk of low adherence. Provision of cCBT may be complemented, for example, by the inclusion of collaborative care protocols to provide scheduled patient follow-ups.^26^

Analysis of the qualitative data was performed prior to analysis of effectiveness in the trial, and was intended to explore patient experiences rather than provide possible explanations for the trial result. Nevertheless, the emphasis of the participants on their struggle to engage with the packages in the absence of support may be one of the key factors responsible for the lack of effectiveness observed in the trial. The discrepancy between the design of the intervention, which incorporated remote support, and patient experience of the treatment as ‘unsupported’ indicates that patient expectations regarding the nature and intensity of support must be addressed when designing low intensity interventions. Expectations of support may also be responsible for the perception of cCBT as inappropriate for more severely depressed patients. A recent individual patient data meta-analysis^27^ suggested that in fact more severe patients could still show demonstrable benefit from minimal interventions. It may be that those patients experiencing more severe depression perceive a greater need for motivational or emotional support to manage their interactions with computerised therapy. Participant reflections also indicate that in such cases the feasibility and appropriateness of including more enhanced support within routine primary care consultations should be addressed. It should be acknowledged however that the limited involvement of GPs in supporting use of the package reported in the present study may be an artefact of the trial context, with GPs assuming that patients were already monitored by the study team. Research under more naturalistic conditions is necessary to address the capacity for GP involvement in supporting engagement with computerised therapies in routine care.

### Unanswered questions and future research

cCBT is unacceptable to a significant proportion of patients as a standalone intervention with minimal support. Research is needed to identify the optimum balance between self-delivered and fully guided treatments in order to enhance acceptability while maintaining cost-effectiveness. Our findings indicate three key questions for future cCBT delivery. First, how to better identify which patients are most or least likely to accept cCBT. Current NICE guidelines account for patient characteristics in recommending antidepressants, and equivalent guidance is needed for self-help treatments.^28^ This would be consistent with calls to provide personalised medicine for depression^29^ to meet both policy targets of patient-centred care and to improve treatment adherence.^30^ Second, research is needed to explore how modifications to computerised treatments could make them more acceptable to currently ambivalent patients drawing on the expertise of disciplines such as Human Computer Interaction^31^ ^32^ to identify how programmes can provide users with a more positive experience and to exploit the potential for greater personalisation of content to encourage adherence. Finally, research should explore the potential for blended protocols (where computerised therapy is supplemented with support from a person) to enhance engagement, considering for example whether such support can be provided remotely or asynchronously and determine whether the cost and accessibility advantages of computerised therapies can be maintained with the provision of human input.
